# The synergistic effect of photobiomodulation, methylglyoxal, and complex magnetic fields on human dermal fibroblasts: potential applications for chronic wound treatments.

**DOI:** 10.1007/s10103-025-04775-3

**Published:** 2025-12-11

**Authors:** Emira D’Amico, Tania Vanessa Pierfelice, Loredana D’Ercole, Paola Di Fermo, Giovanna Iezzi, Simonetta D’ercole, Morena Petrini

**Affiliations:** 1 Department of Medical, Oral and Biotechnological Science, University of Chieti-Pescara, Chieti, Italy; 2 Department of Medical Physics, Fondazione IRCCS Policlinico San Matteo, 27100, Pavia, Italy

**Keywords:** Photobiomodulation, Magnetic fields, MGO, Regeneration, Derma

## Abstract

This paper aimed to verify how a new protocol, recently proposed for treating chronic wounds due to its excellent antimicrobial properties, affects human dermal fibroblasts (NHDFs). Single and combined action of light-emitting diodes (LED), complex magnetic fields (CMFs), and methylglyoxal (MGO) on cell viability and activity of NHDFs were investigated. Our first objective was to exclude any toxicity of this combined treatment on these cells. NHDFs were exposed to LED light for 17 min, CMFs for 22 min, MGO, MGO + LED, and MGO + CMFs, and then were assessed for cell viability, morphology, cytoskeletal integrity, collagen type I production, and migration capacity. Results of combined treatments were compared with those of single treatments and unexposed controls. NHDFs exposed to both single and combined treatments maintained viability, morphology, and cytoskeletal integrity, showing no signs of cytotoxicity. MGO at low concentrations was non-toxic and, when combined with other technologies, could confer beneficial effects on cell adhesion. LED stimulated collagen type I synthesis, and the production increased in samples subjected to the combined action of MGO + LED. CMFs notably accelerated fibroblasts’ migration in scratch assays, and when combined with MGO, they further enhanced this effect. The effects of MGO + LED and MGO + CMFs were probably due to cellular uptake and receptor sensitivity. The tested protocols were not only non-toxic but also promoted beneficial effects on the vitality and activity of dermal fibroblasts, confirming their potential in treating chronic wounds.

## Introduction

Chronic wounds are lesions of the skin that fail to progress through the physiological phases of healing in an orderly and timely manner. These wounds represent a significant clinical challenge due to their persistent inflammation, high susceptibility to infections, and impaired tissue regeneration [1].

Conventional treatments for chronic wounds include debridement, infection control, moisture-balancing dressings, compression therapy, and supportive therapies like growth factors, negative pressure wound therapy, and bioengineered skin substitutes. However, these treatments present various limitations, including high cost, limited effectiveness in complex cases, pain, prolonged sessions, invasiveness, and inability to fully address underlying systemic issues or cellular dysfunctions such as fibroblast senescence [1]. In addition to canonical treatments, innovative and effective strategies have been tested. Among potential therapeutic options, novel technologies and natural compounds have been proposed.

Photobiomodulation (PBM) is a non-invasive treatment that utilizes low-dose light irradiation to promote tissue repair, reduce inflammation, and alleviate pain [2, 3]. Numerous research investigations have reported that PBM accelerates wound healing [4, 5]. Light-emitting diodes (LEDs) are an effective additional treatment method for chronic wounds in people with diabetes in various in vivo studies [2, 6]. In particular, red-light photobiomodulation has been studied for its potential to enhance cell proliferation, migration, and collagen synthesis, which are essential for wound repair [7, 8]. Several studies have demonstrated the antibacterial effects of red-light irradiation at 630 nm against both gram-positive and negative bacteria [9–13]. Among the other potential alternatives for chronic wounds, cellular models exposed to electromagnetic fields (EMFs) showed various biological processes, including the induction of anti-inflammatory pathways and the reduction of reactive oxygen species (ROS) [14]. Complex magnetic fields (CMFs) are composed of EMFs signals of different frequencies, intensities, pulses, and waveforms. The CMFs device is characterized by different programs consisting of a sequence of small, single steps of magnetic fields (3–5 min each), with frequencies ranging between 6 and 70 Hz, intensities between 6 and 95 microT, and complex waveforms with multiple harmonics [15].

Some of these programs have been tested in different conditions [15–17]. These studies have shown a relevant anti-virulence action against *C. albicans* and no cytotoxicity effects on human gingival fibroblasts [15–17]. Zanotti et al. demonstrated the antioxidant, anti-inflammatory, and wound-healing potential of CMFs [18].

Among natural molecules, methylglyoxal (MGO), contained in Manuka honey, has been recently investigated for its anti-inflammatory and antimicrobial properties [19]. MGO is effective against various microorganisms, including some strains of Gram-positive bacteria and certain Gram-negative bacteria, and *C. albicans* [19, 20]. However, the effects of MGO on mammalian cells require careful evaluation due to its possible cytotoxicity. In literature, studies have shown that MGO affects cell viability, proliferation, and apoptosis in a dose-dependent manner [21, 22]. Lee JH. et al., observed that at high concentrations (0.6–1 mM) MGO can induce apoptosis by triggering oxidative stress, mitochondrial dysfunction, and DNA damage in various endothelial cells [22]. Zhang X. et al. described a cytotoxic effect in human umbilical vein endothelial cells (HUVECs) exposed to 0.8 mM of MGO for 5 h. MGO treatment resulted in p53 phosphorylation, cell cycle arrest, and induction of autophagy [21].

Although these innovative strategies are potentially promising, they are still far from completely solving the problem of chronic wounds. Indeed, PBM has been used in medicine for the past three decades, it is still a developing form of therapy, and exploring the combination of PBM with molecules like MGO might be a promising and potentially impactful direction for research and therapy development.

Despite encouraging results, none of these therapies alone is universally recognised for the treatment of chronic wounds. The ideal treatment should promote antibacterial effects while also inducing tissue regeneration, thereby minimizing cytotoxic effects. A recent study demonstrated that the combination of MGO with red PBM and CMFs yielded a more potent antimicrobial effect against *Staphylococcus aureus*,* Pseudomonas aeruginosa*, and *Candida albicans* compared to single treatments alone. These approaches may exhibit considerable potential as a treatment for chronic wounds; however, infection control and the progression of wound healing are equally paramount in the management of these lesions [20]. Beyond its antimicrobial properties, a treatment should not exhibit undesirable effects on the cells responsible for tissue healing and turnover. Thus, this study aimed to investigate the effects of single and combined MGO with red PBM and CMFs on normal human dermal fibroblasts (NHDFs).

## Methods

### Cell culture

Normal human dermal fibroblasts (NHDFs) were purchased by Sigma Aldrich (Darmstadt, Germany) and were cultured with low glucose Dulbecco’s Modified Eagle Medium (DMEM) enriched with 10% of Foetal Bovine Serum (FBS), 1% of penicillin-streptomycin, 1% of L-glutamine (Corning, New York, USA) and 1 ng/mL Fibroblast Growth Factor (FGF) (Sigma Aldrich) at 5% CO_2_ and 37 °C.

### Treatment conditions

#### MGO

Methylglyoxal solution 40 wt% in H_2_O (Sigma-Aldrich, Milan, Italy) at the concentration of 16 µg/mL. The MGO concentration was chosen based on the results of a previous study [20]. For the cytotoxicity test, MGO was added to the cell culture media at increasing concentrations, ranging from 16 µg/mL to 11,000 µg/mL, to determine at which concentration this molecule exhibits cytotoxicity.

#### CMFs

CMFs were generated using the *Next SX* device (Medicina Fisica Integrata, Rome, Italy), which produces pulsed electromagnetic fields between 0.1 and 250 µT and 1–250 Hz. The system includes coils (110 mm external diameter, 12 mm internal diameter, 8 mm thickness) made of 650 turns of 0.35 mm enameled copper wire, positioned at a 90° angle to the sample surface. Among the different operational programs available, the *Antibacterial* program was selected, delivering multi-frequency magnetic fields (6–70 Hz, 6–95 µT) for 22 min, as described in a previous study [20].

#### PBM

**Fig. 1:**
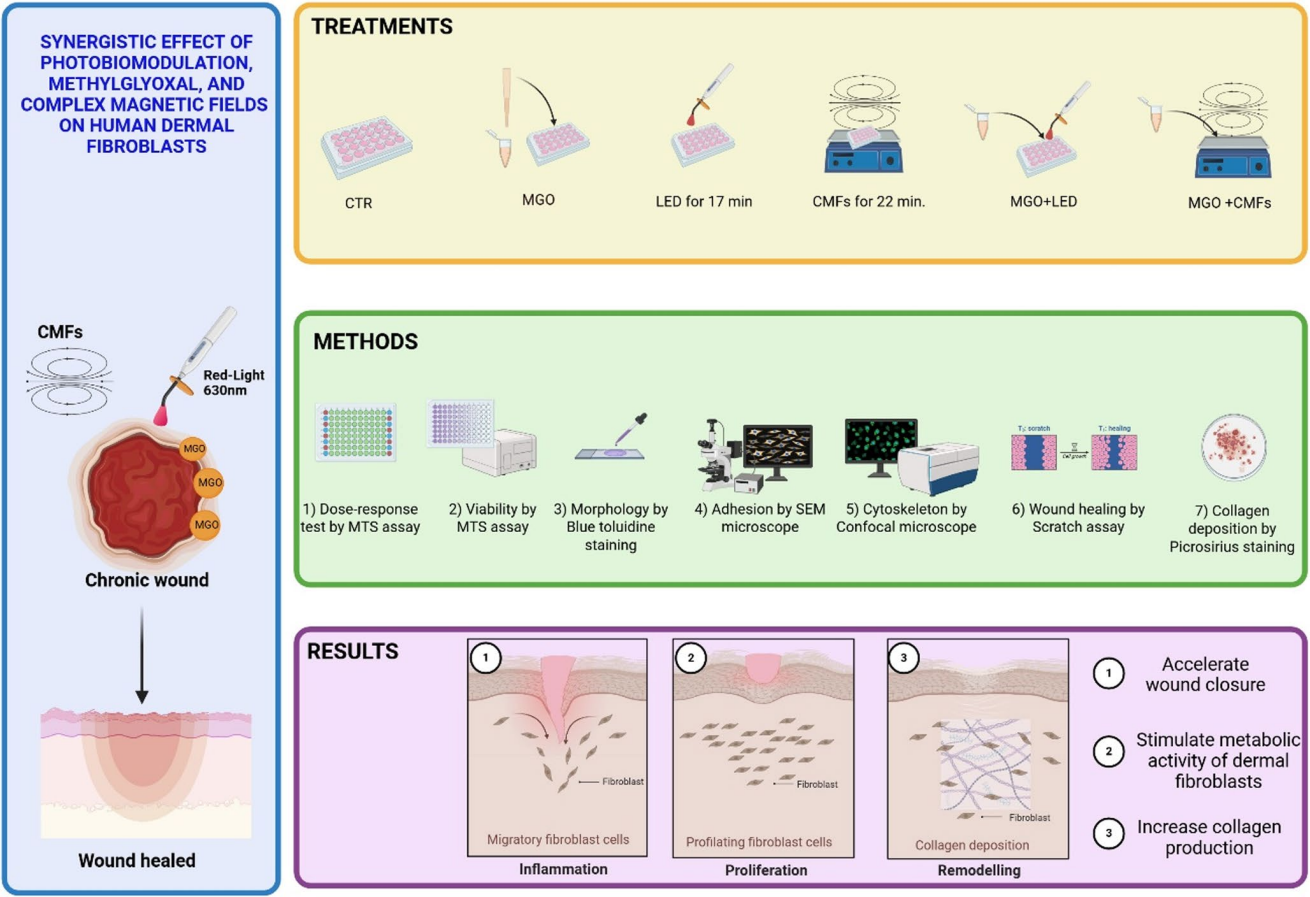
Summary of the material and methods and the principal findings (realized by BioRender.com)

Irradiation with red light was applied using an AlGaAs power LED device (TL-01; ALPHAStrumenti s.r.l., Pero (MI), Italy) characterized by a wavelength of 630 nm, an intensity of 380 mW/cm², and a light dose of 23 J/cm², as previously described [9]. During PBM treatment, each irradiated well was carefully isolated to prevent light diffusion to neighbouring wells, covering the non-irradiated wells with an opaque black material. The LED hand-piece was mounted perpendicularly to the wells at a distance of 0.5 mm with a particular polystyrene box to maintain a constant distance from the light source. The non-irradiated wells were covered with an opaque black material. The time of irradiation was established at 17 min based on previous studies [13, 20].Untreated and unexposed cells were considered as control (CTR). The summary of the material and methods and the principal findings was shown in Fig. 1.

### Evaluation of cytotoxicity and cell viability

10^4^ cells/well were seeded in 96-well plate. Then, NHDFs were treated as explained in the experimental design section, and the viability at 24 h was determined using the MTS assay (Promega, Madison, Wisconsin, USA) according to the manufacturer’s instructions.

### Evaluation of cell density and shape

Cell density and shape of NHDFs were evaluated by toluidine-blue staining. 2 × 10^4^ cells/well were cultured in a 24-well plate and treated as described in the experimental design section. After 24 h, NDHFs were fixed with 70% cold ethanol and stained with toluidine blue. Then, cells were observed using an optical microscope (Leica, Wild Heerbrugg, Wetzlar, Germany) at 3x and 25x magnification.

### Immunofluorescence

NHDFs were cultured in 8-well culture glass slides (Corning, Glendale, AZ, USA) at a density of 1.3 × 10^4^/well and treated according to the experimental design for 24 h. Cells were fixed with 4% paraformaldehyde (PFA) (BioOptica, Milan, Italy) in 0.1 M PBS (Lonza, Basel, Switzerland). Then, the cells were washed three times with PBS and permeabilized with 0.1% Triton X-100 (BioOptica) in PBS for 5–6 minutes. The cytoskeleton actin and the nuclei have been stained, respectively, with rhodamine-phalloidin (Invitrogen) and DAPI (4’, 6-Diamidino-2-phenylindole dihydrochloride; Sigma), both prepared 1:1000 in PBS and maintained for 1 h at 37 °C. The images were acquired through the Zeiss LSM800 confocal system (Carl Zeiss, Jena, Germany).

### Cells count, surface area determination, and nuclei to plasma ratio

Cell surface area (SA) was determined to characterize how cells spread on the surface under given conditions. This value describes the average surface area occupied by cells. Images of fluorescently stained cells were binarised using ImageJ’s tools. From these images, the surface area (µm^2^) occupied by cells was determined with ImageJ software. Next, cell nuclei were counted to obtain the number of cells (n°) using ImageJ software [23]. This software was used to create a binary mask of the nuclei or cytoplasm, to measure the Nucleus to cytoplasm (N/C) ratio. Next, the effective area (%) of the total nuclei was divided by the effective area (%) of the total cytoplasm of cells [23]. Five images, acquired during three repetitive experiments, were analyzed per condition.

### SEM observation

The adhesion capability of cells was tested using scanning electron microscopy (SEM). 10^4^ cells/well were seeded on titanium surfaces (Implacil, DeBortoli, São Paulo, Brazil) as a support [15]and treated as described in the experimental design. Notably, titanium discs have been used instead of plastic culture surfaces because titanium provides a stable and conductive substrate suitable for SEM observation. After 24 h, samples were fixed with 2.5% glutaraldehyde for 1 h, dehydrated using increasing concentrations of ethanol and sputtered with gold. A SEM (Phenom-World B.V., Eindhoven, The Netherlands) was used to observe the samples at 245x and 1000x magnification.

### Wound healing assay

3.5∙10^4^ cells/well were cultured in 24 well-plates until the confluence was reached. Then, a scratch was made in each well using a 200 µl pipette tip, and NDHFs were subjected to the treatment. The wound areas were acquired with a camera connected to an inverted optical microscope (Leica) at 4x magnification at 0, 24, and 48 h. The wound areas, expressed as a percentage, and the migration rate (µm/h) were measured using the software ImageJ 1.52q (National Institutes of Health, Bethesda, MD, USA).

### Picrosirius red staining and spectrophotometric analysis

NHDF cells were cultured in 24-well plates at a density of 5∙10^4^ cells/well and exposed to all treatments. After 7 days, cells were fixed with 2.5% glutaraldehyde for 2 h, incubated with the picrosirius red staining (Sigma Aldrich) at room temperature for 1 h, and images were captured using a stereomicroscope (Leica) at 25×. Then, cells were subjected to three rounds of 0.1% acetic acid washing and to 0.1 N sodium hydroxide. The spectrophotometrical analysis was performed by reading the optical density (OD) at 540 nm.

###  Statistics

All experiments were performed in triplicate, so for each experimental group/condition the biological sample size was 3. Statistical analysis was performed using GraphPad 5 (GraphPad, San Diego, CA, USA) software. One-way analysis of variance (ANOVA) and Tukey’s post hoc test were used to evaluate the differences between groups and intragroup analysis at different time-points. For the quantitative analysis of the scratch assay, two-way ANOVA with Tukey’s post hoc test was applied. A p-value of less than 0.05 was considered statistically significant.

## Results

### Evaluation of MGO cytotoxicity

**Fig. 2:**
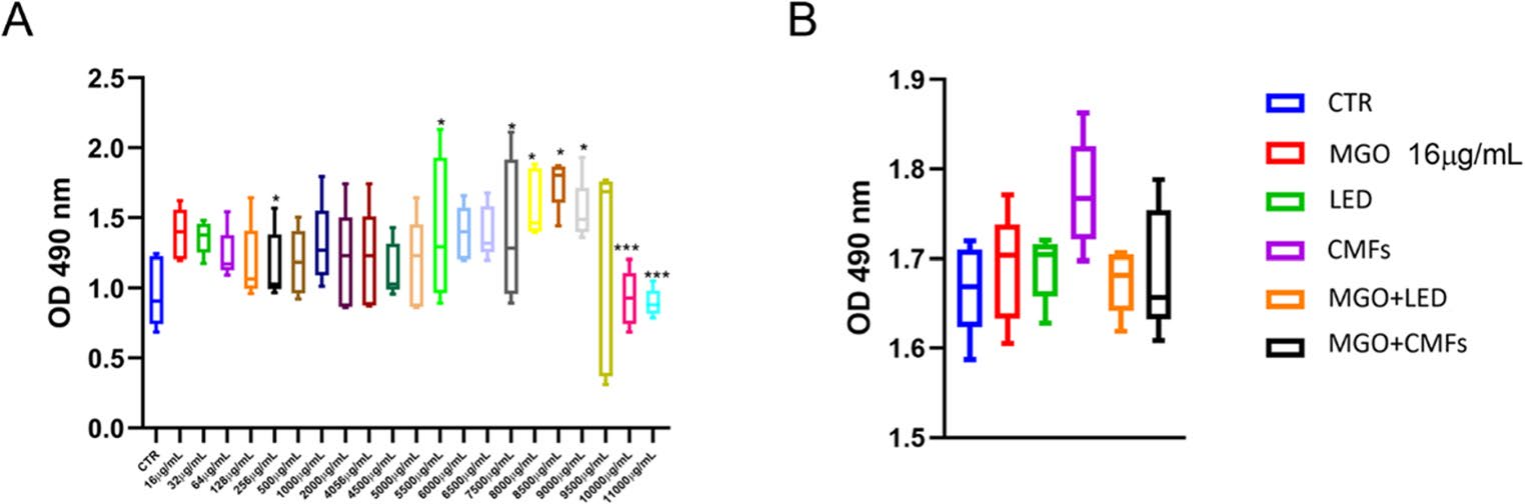
(**A**) Boxplots illustrating MGO cytotoxicity by MTS assay at 24 h. Results were expressed in the form of optical density (OD) at 490 nm. (*p < 0.05; ***p < 0.0001). (**B**) Boxplots illustrating the viability of NHDFs exposed to MGO, LED, and CMFs as single treatments and combined at 24 h. Results were expressed in the form of optical density (OD) at 490 nm. No statistically significant differences were found with respect to unexposed CTR

A variable percentage of viability, ranging from 95% (OD 1,39 ± 0.18) to 130% (OD 1,7502 ± 0.18), was observed after treating NHDFs with MGO at concentrations from 16 µg/mL to 9500 µg/mL at 24 h (Fig. 2A). Only at the concentrations of 10,000 µg/mL and 11,000 µg/mL, a severe decrease in the viability was observed, obtaining values of ~ 50%. These values appeared significant compared to CTR (*p* < 0.0001).

### Viability of dermal fibroblasts

The viability of NHDFs exposed to treatments such as MGO 16 µg/mL, LED, CMFs and MGO + LED showed a cell growth higher than untreated cells (CTR) at 24 h. Whereas NHDFs exposed to MGO + CMFs showed a slight decrease of viability with respect to control. However, any differences between treated groups (MGO, LED, CMFs, MGO + LED and MGO + CMFs) and control group (CTR) resulted no statistically significant. (Fig. 2B).

### Cell density, morphology, and adhesion

**Fig. 3:**
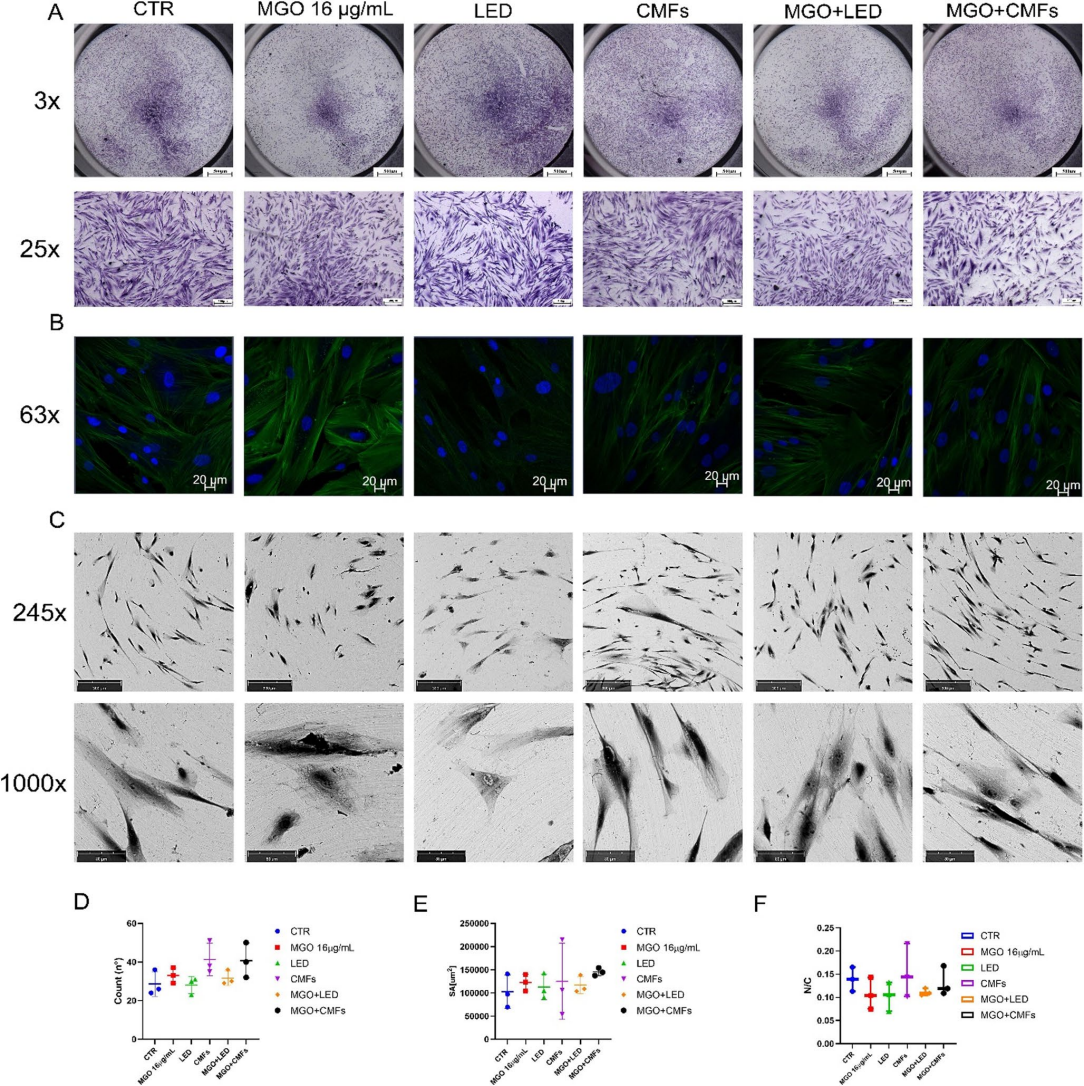
(**A**) Toluidine blue staining observations at the optical microscope at 24 h. (First line: magnification 3x, scale bar: 500 μm; Second line: magnification 25x, scale bar: 300 μm). (**B**) Confocal laser scanning microscope (CLSM) images showing DAPI staining (blue fluorescence), which highlights cell nuclei, and phalloidin–rhodamine staining (green fluorescence), which visualizes the cytoskeleton. Magnification: 63×; scale bar: 20 μm. (**C**) SEM observations, (Fourth line: magnification 245x; scale bar: 300 μm Fifth line: magnification 1000x, scale bar: 80 μm). (**D**) Count (n°) of cells (Box plot denotes an average number of cells in the images). (**E**) Spreading area (SA) of cells. Box plot denotes an average surface area of cells in the images. **(F**) Nucleus to cytoplasm (N/C) ratio of cells. (Box plot denotes an average surface area of cells in the images). Boxplots D, E,F represent basic statistical parameters (mean, median, standard deviation, and 25% and 75% percentiles from *n* = 5 fluorescent images). Statistical significance: not statistically significant (*p* > 0.05)

Cells exposed to the treatments were very similar to those of untreated CTR in their morphological features, density, and surface adherence. All groups maintained high cell viability, and cell density appeared preserved as observed in Fig. 3A at 3x and 25x, together with the structure of nuclei and cytoskeletal filaments (Fig. 3B). Also, at the SEM, all exposed cells maintained their original shape observed at 245x. Numerous interconnections and filopodia among cells were observed at 1000x (Fig. 3C). The combined action of MGO + LED and MGO + CMFs promoted a slight increase in cell adherence to the titanium surfaces, respect single treatments and unexposed controls. The most notable increase in cell number was observed in cells treated with CMFs, particularly in the combined MGO + CMFs (Fig. 3D). Treated cells displayed different surface areas, indicating varying abilities to spread on the surface. A larger surface area corresponds to greater adhesion. The most marked change was observed in MGO and in MGO + CMFs (Fig. 3E). N/C ratio decreased in all treated cells, except for those treated with CMFs, whose values were similar to those of the control group (Fig. 3F).

### Wound healing potential

**Fig. 4:**
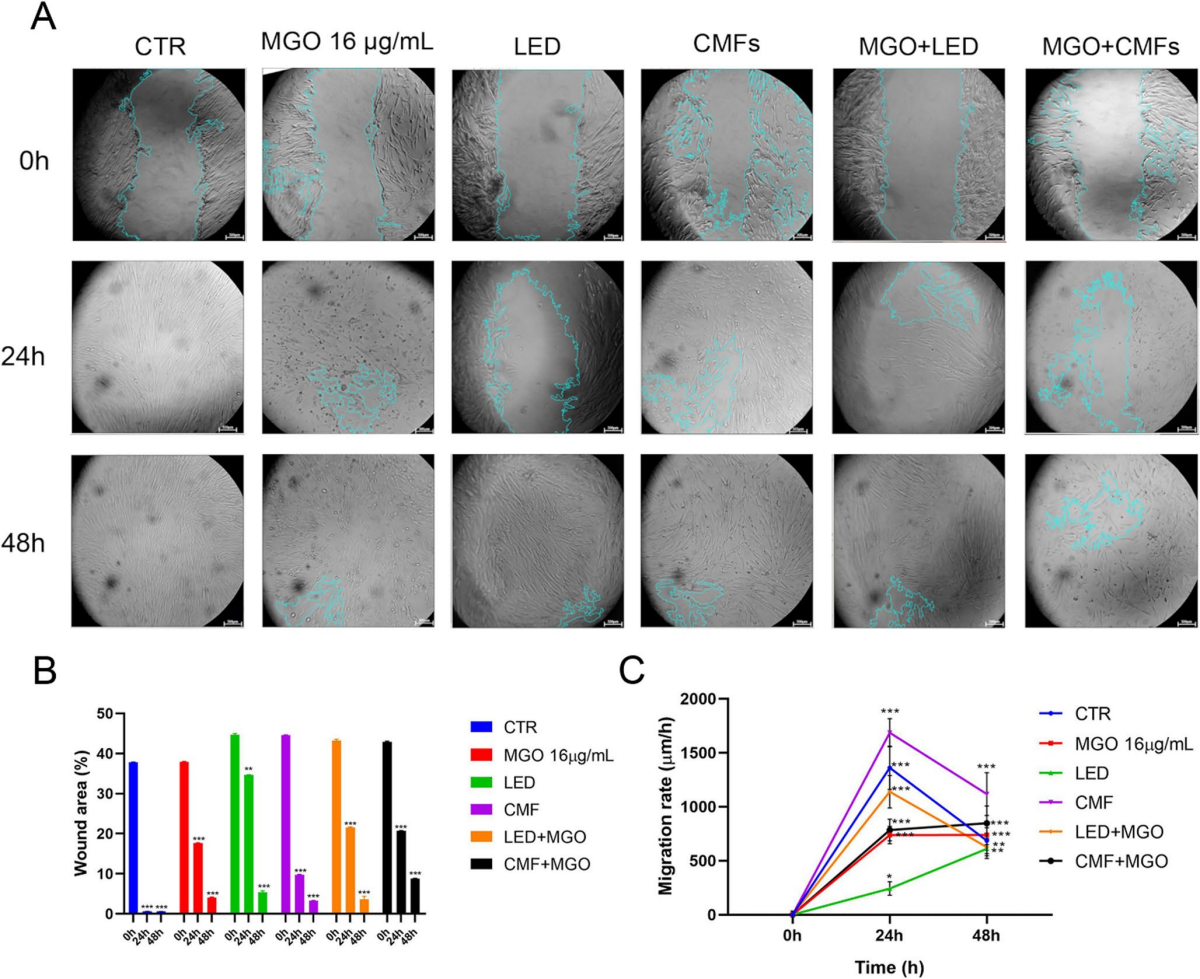
(**A**) Scratch assay of unexposed and exposed groups at different timings: 0, 24, and 48 h. The unclosed area has been highlighted in blue (magnification 4x, scale bar: 300 μm). The comparisons are shown with respect CTR at t0. (**B**) Percentage of wound area and (**C**) migration rate calculated on the scratch assay. (** *p* < 0.001; ***p < 0.0001). The comparisons are shown with respect CTR at t0

All groups showed a progressive reduction in wound area over time, indicating active cell migration and wound closure (Fig. 4A). The graph quantifying wound area (µm²) over time (Fig. 4B) showed that at 0 h, all groups started with comparable wound areas, confirming uniform scratch creation across conditions. At 24 h, CTR had the smallest wound area (0.64 ± 0.02%). All exposed groups showed a statistically significantly larger wound area at 24 h compared to CTR (*p* < 0.001). Among treatment groups, MGO + CMFs showed a significantly larger area than the single actions of MGO and CMF alone (*p* < 0.001), while MGO + LED performed better than LED alone, suggesting a synergistic effect. At 48 h, wound areas decreased further in all groups. CTR maintained the smallest wound area (0.59 ± 0.03%), with statistically significant differences compared to all treated groups (*p* < 0.001). Among treatment groups MGO + LED showed improved closure compared to LED alone (*p* < 0.001), but no significant difference compared to MGO. MGO + CMFs remained the least effective, with the highest residual wound area (8.75 ± 0.16%), followed by LED, MGO (4.08 ± 0.09%), MGO + LED (3.61 ± 0.80%), and CMFs (3.26 ± 0.09%). The migration rate of NHDFs was quantified at 0 h, 24 h, and 48 h. All groups started at 0 μm/h at 0 h, confirming uniform scratch creation (Fig. 4C). At 24 h, all treated groups exhibited increased migration rates, with the highest value observed in the MGO + CMFs group, followed by MGO + LED, and CMFs. The lowest rates were recorded in MGO and LED groups. CMFs significantly enhanced migration compared to MGO (*p* < 0.001) and LED (*p* < 0.001). The combination of MGO + CMFs significantly outperformed MGO alone (*p* < 0.001) and LED (*p* < 0.001), suggesting a synergistic effect on cell motility. At 48 h, migration rates generally declined across all groups, consistent with the progression toward wound closure. Despite the reduction, MGO + CMFs maintained the highest migration rate, followed by CMFs, MGO + LED, and CTR. No statistically significant differences were observed between combined and single treatments at this time point, indicating convergence of migration dynamics. 

### Collagen production

**Fig. 5:**
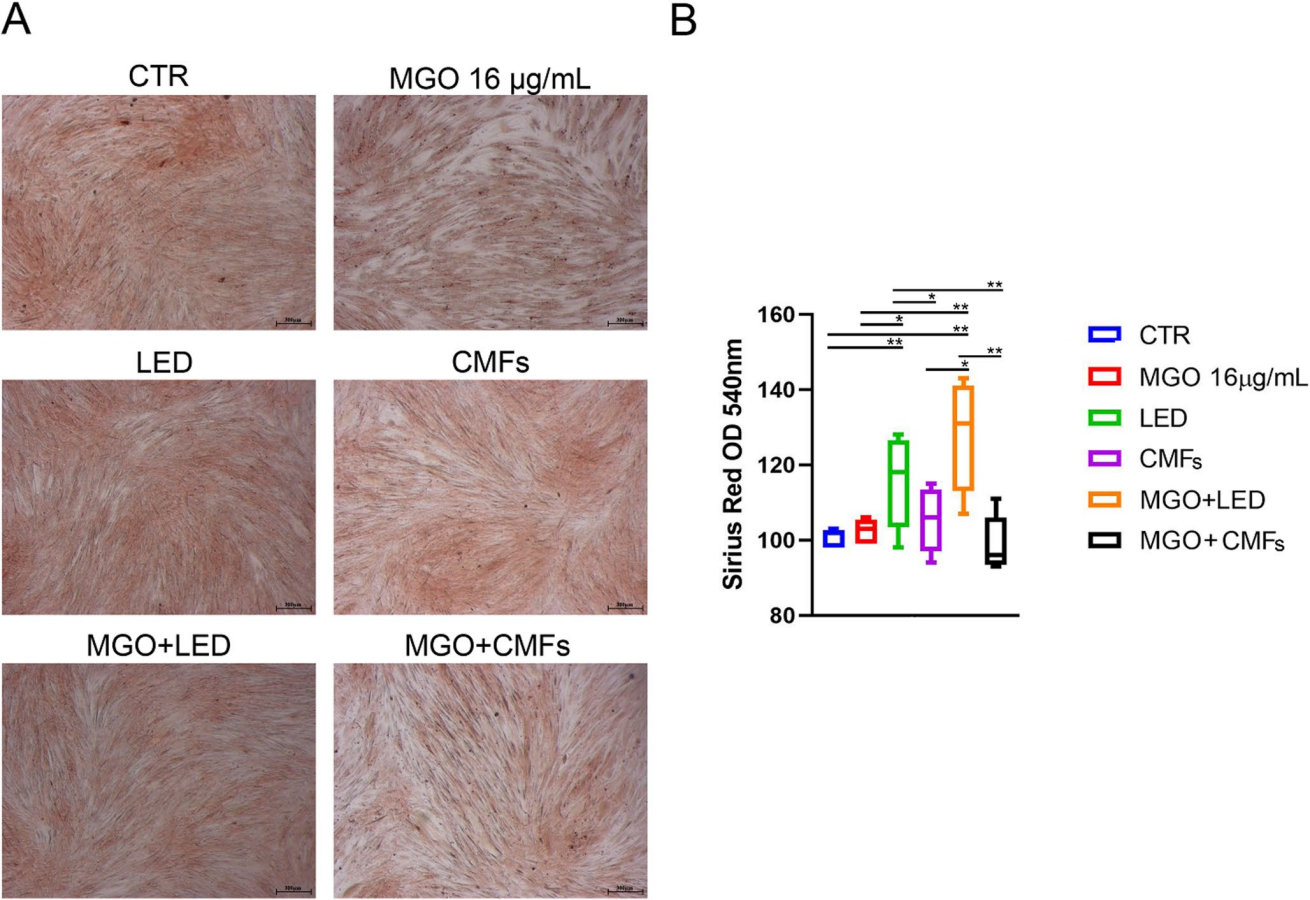
Picrosirius red staining of CTR and exposed groups observed at the optical microscope, magnification: 25x; scale bar 300 μm (**A**). Sirius-Red staining quantification (**B**) at spectrophotometric analysis, expressed as optical density (OD) (*p < 0.05; **p < 0.001)

Picrosirius red staining revealed more intense red deposits after irradiation with LED and MGO + LED compared to other treatments (Fig. 5A). The spectrophotometric analysis confirmed the qualitative evaluation with quantitative measurements. In detail, the MGO + LED condition exhibited the highest collagen deposition, with a significant increase in OD values compared to CTR (*p* < 0.001), MGO (*p* < 0.001), CMFs *(p* < 0.005), and MGO + CMFs *(p <* 0.05).

LED also showed moderate increases in collagen deposition, and no significant differences were found respect MGO + LED. On the contrary, significant differences *(p* < 0.001) were found at the intergroup analysis between LED and CMFs, MGO, MGO + CMFs, and CTR. Listing the groups in descending order of collagen production, the greater values were found for MGO + LED and LED, followed by CMFs, MGO, MGO + CMFs, and CTR. No significant differences were found between CMFs, MGO, MGO + CMFs, and CTR (Fig. 5B).

## Discussion

The combined action of MGO + LED, and MGO + CMFs demonstrated significant potential against chronic wounds due to their antimicrobial effects. However, there is a gap in the literature about the potential effects of these combined strategies on NHDFs. Considering the impact of the single treatments, MGO at the dose used by Diban et al. [20], 16 µg/mL, showed no cytotoxicity against NHDF. In particular, this molecule has shown no cytotoxicity up to a concentration of 10,000 µg/mL, indicating that it remains safe at or below this dosage. In the literature, the effects of MGO remain somewhat controversial. Several in vitro studies have demonstrated that MGO, at concentrations between 0.6 and 1 mM, can inhibit cell growth by inducing apoptosis [24, 25]. Conversely, an in vivo study found that MGO did not produce harmful effects on the vital organs of various animal models [26]. Additionally, other single treatments, such as CMFs and LED, did not affect the viability of NHDFs. These results were in line with previous literature, which indicates that pulsed electromagnetic fields (PEMF) enhance early wound healing and promote myofibroblast proliferation in diabetic rats [27]. Vinck et al. reported positive effects of PBM, using various wavelengths (950 nm, 660 nm, and 570 nm), on fibroblast proliferation [28]. Jere et al. recently reported a significant increase in migration and proliferation of normal, wounded, diabetic, and diabetic-wounded WS1 fibroblast cells when irradiated with a 660 nm diode laser at a fluence of 5 J/cm² [29]. Surprisingly, the combination of 16 µg/mL MGO + CMFs, and MGO + LED in the study of Diban et al. was characterized by a remarkable antimicrobial activity, in terms of viable cell count, motility, and cellular membrane permeability and fluidity, against *Candida albicans* and *Pseudomonas aeruginosa*, and did not affect NHDFs’ proliferation [20]. The absence of cytotoxicity of this protocol was tested in this study and confirmed through optical, confocal, and scanning electron microscope observations, which showed the typical spindle-shaped morphology and cytoskeleton of normal fibroblasts in both exposed and unexposed cells.

The observed thickening of actin filaments in MGO-treated cells suggested a response to a possible stress induced by this molecule.This might reflect cytoskeletal remodeling in response to the glycation stress induced by MGO [30]. Interestingly, this effect was not accompanied by significant morphological alterations in actin filament organization, although an increase in filament thickness. The combined treatments (MGO + LED and MGO + CMFs) appeared to mitigate MGO-induced effects, maintaining actin architecture and spreading areas similar to control cells. This may suggest a protective or modulatory role of LED or CMFs against MGO-induced cytoskeletal stress, potentially by influencing signaling pathways involved in cytoskeletal dynamics or oxidative stress responses. N/C ratio decreased in treated cells, except for those treated with CMFs alone, which maintained control-like values. A lower N/C ratio typically indicates increased cytoplasmic volume, potentially reflecting enhanced cell spreading or cytoplasmic reorganization [23, 31]. This finding aligns with the surface area measurements and supports the hypothesis that CMFs may promote or preserve cytoplasmic integrity and cell adhesion capacity under stress conditions. Indeed, in the initial stages of wound healing, preserving cytoskeletal integrity enables fibroblasts to migrate to the wound site and deposit extracellular matrix, thereby reducing treatment-related stress [32]. Photobiomodulation seemed to delay wound closure, as evidenced by partial closure at 24 h. There is insufficient evidence of the effects of red LED irradiation on scratch assay closure on NHDFs after 24 h. Theodoro et al. showed that red LED irradiation at 635 nm, 1.45 J/cm² of NIH/3T3 fibroblasts had no significant differences compared to untreated controls regarding the proliferation and migration rate during the first 4 days of observation [33]. However, after 48 h, the wound closure in the LED group increased significantly and was nearly complete, confirming that LED irradiation promoted wound closure, even though the migration rate was lower than that of untreated cells. This finding was in agreement with a recent study indicating that the cell migration was unaffected until 12 h upon 630 nm LED treatment and started to accelerate after 24 h [34]. MGO at the concentration used in this study did not seem to affect wound closure, although other concentrations between 7.5 and 10 mM MGO led to enlarged scratch areas in NHDF after 26 h, indicating a significant decline in cell migration and viability [35]. The study of the wound area showed that at 24 h, the combined action of MGO + LED produced better results than LED alone, but worse results than MGO alone. Conversely, at 48 h, the combined action showed better results than the single treatments, but with significant results only compared to LED. On the contrary, the combined action of MGO + CMF showed worse results compared to the single treatments, both at 24 and 48 h. Migration rate, quantified by dividing the change in wound width by the time spent in migration, was highest in CMFs-treated cells. The application of CMFs seemed to accelerate the migration rate of MGO-treated cells. Indeed, the combined action of MGO + CMFs showed a significantly higher migration rate compared to MGO alone, at 24 h. Notably, the increased cell counts and stronger adhesion observed in the MGO + CMFs group do not directly equate to enhanced wound closure: stronger adhesion and increased spreading can reduce migratory velocity at the wound edge, so proliferation and adhesion changes may not contribute to scratch closure. The contradictory findings of various studies on MFs suggest that combinations of intensity and treatment period may produce different effects on extracellular matrix synthesis and remodelling, cell proliferation, and migration [36, 37]. The combination of MGO + LED stimulated a better wound closure than LED and MGO alone, at 24 h. At 48 h, no significant differences were found between single and combined action for the migration rate. The remodelling phase of chronic wound healing involves collagen synthesis [38]. In the present study, LED-irradiated cells produced significantly more collagen than untreated cells. In particular, MGO + LED improved the effects of MGO in the synthesis of collagen by fibroblasts. On the contrary, the combination of MGO + CMFs did not increase the collagen deposition, with respect to CTR, MGO, and CMFs alone. Literature describes the beneficial effects of light therapy on collagen production. A histology study reported differences in collagen content in an animal model. It showed that collagen fibers were more organized in diabetic and non-diabetic rats after low-level laser therapy (904 nm) compared to untreated ones [39]. The effects of LED on both migration and collagen synthesis are highly dependent on wavelength, fluence, and treatment protocol, and the effect on fibroblasts can persist up to 21 days after irradiation [40]. In this study, LED irradiation seems to decrease wound closure and migration rate with respect to controls at 24 h, but at 48 h, the same cells increased their values, reducing the differences with other groups. Moreover, at 7 days, the collagen production was higher in the LED group, confirming that this treatment stimulated cell activity and the effects persisted for more days after irradiation. In this study, each combined treatment exerted beneficial effects on cells. MGO at low concentrations was not cytotoxic for dermal fibroblasts; for this reason and due to its antibacterial properties, it might be used as MGO-based dressings to treat many types of wounds, including chronic wounds. The eco-friendly technologies CMFs seemed to be a promoter of the proliferation and cell migration that characterize the initial phase of the healing process. In contrast, LED appeared to stimulate the deposition of collagen type I, which is crucial in the late stage of the healing process. Dang et al. observed that a wavelength of 800 nm and a fluence of 40 J/cm^2^ increased skin collagen synthesis via the Smad pathway [41]. At a wavelength of 670 nm, Otterco et al. observed an improved wound healing process on wounded rats compared to the non-irradiated control group. The authors noted a significant decrease in the inflammatory cytokine TNF-α, and an increase in collagen type I [42]. Fibroblasts are responsible for generating the majority of the extracellular matrix (ECM) during tissue repair, which is crucial for tissue remodeling and the complete closure of wounds. Collagen, a key element of the ECM, is produced and regulated through a balance between its synthesis and degradation by enzymes like matrix metalloproteinases (MMPs). The findings of this study indicate that combining technologies such as CMFs and PBM with molecules like MGO yields greater benefits than using either treatment individually. A possible explanation might be that LED, and CMFs can affect drug transport and cellular receptor sensitivity. It has been reported that MFs can influence ion channels and signaling pathways, thereby altering cell responses to bioactive molecules [43]. LED light can increase cell membrane permeability, microcirculation, and cellular metabolism, which can enhance penetration and cellular uptake of molecules [44]. However, the present study showed limitations. The experiments were conducted exclusively in vitro, which does not fully reproduce the complex physiological environment of chronic wounds, including interactions with immune cells, vascularization processes, and microbial diversity. In addition, this study focused primarily on short-term cellular responses; therefore, evaluating the long-term effects would provide more comprehensive insights into the sustained impact of these treatments.

Further research, especially in vivo studies to validate their clinical relevance, is needed. In the future, the combination of PBM and CMFs may not only be feasible but could also represent a promising and innovative direction in biomedical research. Both PBM and CMFs therapies are highly adaptable and well-suited to personalized medicine, offering the potential for individualized treatment strategies. By adjusting key parameters, such as wavelength, fluence, field strength, and duration, these therapies can be tailored to the specific needs and biological responses of each patient. With further research and clinical validation, the integration of PBM and CMFs could become a powerful and non-invasive approach to precision therapy.

## Conclusion

This study showed that all devices tested, at the parameters described, were not toxic and are safe for dermal fibroblasts, but produce beneficial effects on the cellular activity of dermal fibroblasts, which are mainly responsible for skin wound healing.

## Data Availability

Data is provided within the manuscript, but if other data are necessary, you can contact the corresponding authors.
